# Feasibility of a tele-prehabilitation program in high-risk patients with colon or rectal cancer undergoing elective surgery: a feasibility study

**DOI:** 10.1186/s13741-022-00260-5

**Published:** 2022-07-26

**Authors:** Ruud F. W. Franssen, Bart C. Bongers, F. Jeroen Vogelaar, Maryska L. G. Janssen-Heijnen

**Affiliations:** 1 Department of Clinical Physical Therapy, VieCuri Medical Center, Tegelseweg 210 5912BL, Venlo, the Netherlands; 2 Department of Epidemiology, GROW School for Oncology and Developmental Biology, Faculty of Health, Medicine and Life Sciences, Maastricht University, Maastricht, the Netherlands; 3 Department of Nutrition and Movement Sciences, School of Nutrition and Translational Research in Metabolism (NUTRIM), Faculty of Health, Medicine and Life Sciences, Maastricht University, Maastricht, The Netherlands; 4 Department of Epidemiology, Care and Public Health Research Institute (CAPHRI), Faculty of Health, Medicine and Life Sciences, Maastricht University, Maastricht, The Netherlands; 5 Department of Surgery, VieCuri Medical Center, Venlo, the Netherlands; 6 Department of Clinical Epidemiology, VieCuri Medical Center, Venlo, the Netherlands

**Keywords:** E-health, prehabilitation, Exercise fidelity, Preoperative management, Abdominal surgery

## Abstract

**Background:**

Prehabilitation appears to be an effective strategy to reduce postoperative complications and enhance recovery after colorectal surgery. Although many patients prefer (unsupervised) home-based prehabilitation, adherence can be problematic. Combining home-based prehabilitation with tele-monitoring might demonstrate a higher adherence than unsupervised prehabilitation; however, evidence on its feasibility and effectiveness in patients with colorectal cancer scheduled for elective surgery who are at high risk for postoperative complications is lacking. The aim of this study was to assess the feasibility of a bimodal tele-prehabilitation program in patients with colorectal cancer at high risk for postoperative complications.

**Methods:**

High-risk patients (oxygen uptake at the ventilatory anaerobic threshold ≤11 mL/kg/min or oxygen uptake at peak exercise ≤ 18 mL/kg/min) with colorectal cancer were included in a home-based bimodal tele-prehabilitation program. The program consisted of a personalized tele-monitored moderate to high-intensity interval training intervention and nutritional counseling. Feasibility was measured by participation rate, dropout rate, adherence to the physical exercise training session’s frequency, intensity, and time, and retention rate. Patient appreciation was measured by a patient appreciation questionnaire. Changes in preoperative physical fitness as secondary outcomes were quantified by time to exhaustion on a constant work rate (cycle) test, number of repetitions on the 30-s chair-stand test, and walking speed on the 4-m gait speed test.

**Results:**

The participation rate was 81%, there were no adverse events, and all participants managed to complete the tele-prehabilitation program (retention rate of 100%). Adherence with regard to the exercise program’s frequency, intensity, and time was respectively 91%, 84%, and 100%. All participants appreciated the tele-prehabilitation program. Time to exhaustion on the constant work rate test improved (not statistically significant) from a pre-prehabilitation median score of 317 seconds to a post-prehabilitation median score of 412 seconds (*p* = 0.24). Median number of repetitions on the 30-s chair-stand test improved from 12 to 16 (*p* = 0.01).

**Conclusions:**

Tele-prehabilitation seems feasible in high-risk patients with colorectal cancer, but efforts should be made to further improve adherence to physical exercise training intensity. More research is needed to establish the (cost-)effectiveness of tele-prehabilitation regarding preoperative improvements in preoperative aerobic fitness and postoperative reduction of complications.

**Trial registration:**

ISRCTN, ISRCTN64482109. Registered 09 November 2021 - Retrospectively registered.

## Background

There is a growing amount of evidence showing that prehabilitation can effectively improve preoperative aerobic fitness and reduce the incidence of postoperative complications in patients who are referred for abdominal surgery (Barberan-Garcia et al. 2018) and surgery for colorectal cancer (Berkel et al. 2021; de Klerk et al. 2021) when aiming at patients at high risk for complications. Patients at high risk for postoperative complications after abdominal surgery often have a low aerobic fitness, are physically vulnerable, suffer from multimorbidity, are of older age (van Rooijen et al. 2017), and depend on others for transport (Berkel et al. 2018). Therefore, for high-risk patients, participation in center-based prehabilitation is often difficult (Thomas et al. 2019). Among perceived barriers that hinder patients from participating in prehabilitation are the many hospital appointments (Agasi-Idenburg et al. 2020; Beck et al. 2021), finding time (Berkel et al. 2022; Ferreira et al. 2018), distance from the prehabilitation facility (Woodfield et al. 2018), and transportation issues (Berkel et al. 2022; Ferreira et al. 2018; Prepare-Abc Trial Collaborative 2021).

Evidence from interviews among patients who underwent major abdominal surgery for cancer demonstrated that many patients prefer home-based prehabilitation (Ferreira et al. 2018; Beck et al. 2022). A home-based approach offers safety for patients who experience nausea, diarrhea, or physiological issues (Beck et al. 2022), provides flexibility towards medical/personal commitments (Wu et al. 2021), resolves transportation issues (Wu et al. 2021), and enhances social support (Wu et al. 2021). In addition, home-based prehabilitation enables patients to combine prehabilitation with practical tasks and social activities of everyday life that are perceived as meaningful in the often short and stressful period between cancer diagnosis and treatment (Beck et al. 2022). Considering the abovementioned needs and preferences of high-risk patients, a home-based approach might be desirable.

A disadvantage of (unsupervised) home-based prehabilitation as opposed to supervised hospital-based prehabilitation is that adherence can be problematic without supervision (Lacroix et al. 2017). A systematic review reported mean adherence rates of > 95% in studies evaluating hospital-based (supervised) prehabilitation opposed to only about 70% in studies evaluating (unsupervised) home-based prehabilitation (Thomas et al. 2019). As the preoperative period is often short and time-constrained (2–6 weeks), high-intensity physical exercise training with high exercise training adherence is of major importance for prehabilitation to be effective (Franssen et al. 2022). To improve adherence, prehabilitation should not only be personalized to a patient’s aerobic fitness, everyday activities, and preferences, but should also involve some degree of support and pressure to be motivational (Beck et al. 2021).

By using technologies like tele-monitoring (e.g., tele-prehabilitation,) the benefits of home-based and supervised prehabilitation might be combined. This way, adherence can be measured objectively and accurately, and patients can be coached, motivated, and encouraged via tele-monitoring while performing their home-based individualized training sessions at a time and place of their preference. Evidence in patients with musculoskeletal conditions suggests that, compared to classic unsupervised home-based programs, tele-monitoring can improve adherence (Lambert et al. 2017). To date, a few studies have investigated the feasibility of home-based tele-prehabilitation programs prior to colorectal cancer surgery (Wu et al. 2021; Bruns et al. 2019), and concluded that tele-prehabilitation was feasible, appreciated by patients, and has the potential to improve physical fitness. However, these studies (Wu et al. 2021; Bruns et al. 2019) failed to report full feasibility as adherence to the physical exercise training’s frequency, intensity, and time was lacking. Moreover, none of these studies (Wu et al. 2021; Bruns et al. 2019) specifically included patients at high risk for postoperative complications determined by preoperative cardiopulmonary exercise testing (CPET).

The aim of this pilot study was to investigate whether a home-based and tele-monitored prehabilitation program (tele-prehabilitation) is feasible in high-risk patients scheduled for colorectal cancer surgery. Secondary aims were to evaluate patient experiences and changes in preoperative aerobic fitness before and after the tele-prehabilitation program.

## Methods

### Study design

The current pragmatic one-arm pilot feasibility study was carried out at VieCuri Medical Center, a large teaching hospital in Venlo, the Netherlands. The study was approved by the Medical Ethics Review Committee – Zuyderland/Zuyd (Heerlen, the Netherlands) under reference number METCZ20190150. Initially, the trial started in February 2020; however, due to restrictions caused by the worldwide COVID-19 pandemic, inclusion could only start in July 2020 and ended in September 2021. Reporting was done in accordance with the CONSORT statement extension to randomized pilot and feasibility trials (Eldridge et al. 2016).

### Participants

A consecutive sample of potentially high-risk patients was recruited at the moment of suspected colorectal cancer by endoscopy. A few days after the endoscopy, patients were contacted by telephone to check for potential eligibility and willingness to participate. Patients were potentially eligible when they were ≥ 18 years of age, were able to operate a mobile phone, and had a score ≤ 7 metabolic equivalents of task (METs) on the veterans-specific activity questionnaire (VSAQ). These eligibility criteria were used as a pre-screening. Final eligibility was determined after CPET and final diagnosis, which was defined as an oxygen uptake (VO_2_) at the ventilatory anaerobic threshold (VAT) ≤ 11 mL/kg/min or a valid VO_2_ at peak exercise (VO_2peak_) ≤ 18 mL/kg/min during CPET in combination with confirmed diagnosis of colon or rectal cancer (stage I, II, or III) requiring elective resection with or without neoadjuvant treatment.

### Intervention and assessments

A multimodal tele-prehabilitation program was embedded within the existing colorectal cancer pathway of VieCuri Medical Center. Therefore, no additional hospital visits were required for study purposes. Pre-prehabilitation measurements (*T*_0_) were planned on the day of the appointment with the surgeon, approximately 2–5 days after final inclusion. In patients receiving neoadjuvant treatment, pre-prehabilitation measurements were performed concurrent with the first appointment with the surgeon after completing neoadjuvant treatment (approximately 4 weeks before surgery). Pre-prehabilitation (*T*_0_) assessments consisted of evaluating aerobic fitness by time to exhaustion on a continuous work rate test at 80% of the peak work rate achieved during CPET. Additionally, lower limb muscle power and endurance was assessed by the number of repetitions on the 30-s chair-stand test and gait speed was measured using the 4-m gait speed test. Post-prehabilitation (*T*_1_), reassessment of the continuous work rate test, 30-s chair-stand test, and the 4-m gait speed test took place 1 or 2 days prior to surgery. In addition, participants filled out a patient appreciation questionnaire, based on the questionnaire of Dronkers et al. (Dronkers et al. 2010), and the systems usability questionnaire (Myers et al. 1994) after the post-prehabilitation assessment.

The tele-prehabilitation program consisted of a tele-monitored physical exercise training module and a nutritional support module. Encouraging smoking cessation was part of usual care and was therefore not included explicitly in the tele-prehabilitation program.

#### Physical exercise training

The tele-monitored physical exercise training module was delivered by using the mobile phone application of HC@Home (version HC1.12a, HC@Home B.V., Zwolle, the Netherlands) on a dedicated mobile phone (delivered to the patients for the duration of the tele-prehabilitation program) to which a heart rate monitor (Polar OH1, Polar Electro Inc., Kempele Finland) was connected. Personalized training zones were set based on the heart rate at the VAT and the respiratory compensation point as determined by CPET. Ideally, training sessions took place every other day and consisted of 30 min of aerobic moderate- to high-intensity interval training by a patient’s preferred activity (i.e., walking, cycling, stair climbing, sit-to-stand exercises, push-ups, steps). Intervals consisted of 3 min of low-intensity exercise at a heart rate below the heart rate at the VAT and/or a 6*–*20 Borg rating of perceived exertion (RPE) score ≤ 11, interspersed by 3 min of high-intensity exercise at a heart rate just below the heart rate at the respiratory compensation point (approximately 70*–*85% of the heart rate at VO_2peak_) or a Borg RPE score of 14*–*16. In-between training days, patients were advised to retain relative rest but still comply with the Dutch physical activity guidelines (e.g., > 30 min of moderate-intensity physical activity). The abovementioned training protocol was used as a blueprint, which means that training frequency, intensity, time, type, volume, and progression were personalized according to CPET results (e.g., using shorter intervals in patients with a pulmonary exercise limitation), training heart rate, training Borg RPE score, recovery after training, and participant experiences and preferences. After the first face-to-face physical exercise training at home, which was supervised by a physical therapist specialized in physical exercise training in clinical populations, participants continued the home-based physical exercise training sessions independently. Involvement of a family member or (informal) caregiver during exercising was encouraged to promote motivation. The first face-to-face session was used to validate training zones and familiarize participants with the exercises and equipment. Performed training session’s frequency, intensity, and time were automatically uploaded to an online platform, at which they could be reviewed by the physical therapist. A weekly phone call took place to monitor training progression and adjust the physical exercise program accordingly.

#### Nutritional counseling

Participants were screened for malnutrition using the patient-generated subjective global assessment short form (PG-SGA-SF) in combination with a comprehensive nutritional screening by a registered dietician. Preoperative nutritional counseling consisted of optimization of basic nutritional needs, as well as ensuring the recommended intake of protein, defined as 1.2–2.0 g/kg body mass (Wischmeyer et al. 2018). After an initial intake assessment, follow-up counseling was provided by a weekly phone call between the dietician and the participants in order to monitor nutritional and protein intake, as well as to compare nutritional and protein intake against calculated needs. In addition, body mass was assessed based on self-report and participants were motivated to comply with the dietary advice.

#### Outcomes

The primary outcome of the study was feasibility as determined by (1) study participation rate combined with reasons for non-willingness or inability to participate, (2) the number and severity of adverse events related to the physical exercise training program, (3) adherence to the physical exercise training program, (4) study dropout rate and reasons for dropouts, and (5) retention rate. Secondary outcomes were (1) participant experiences as measured by the patient appreciation questionnaire, (2) user-friendliness of the mobile phone application that was used for tele-prehabilitation assessed using the systems usability questionnaire (Myers et al. 1994), and (3) changes in physical fitness during the tele-prehabilitation program.

#### Statistical analyses

Statistical analyses were performed using IBM SPSS Statistics version 26.0 (IBM, Chicago, IL, USA). Participation rates were reported descriptively as numbers and percentages of the potentially eligible patients that were willing to participate in the current study. Dropout rates and adverse events were reported as numbers and as a proportion of participants enrolled in the study. Retention rate was expressed as a percentage and defined as the proportion of enrolled participants that completed the program. Adherence to the physical exercise training program with regard to training frequency, training intensity, and training time was determined as follows. For training frequency, observed training frequency was divided by the prescribed frequency and expressed as a percentage. Regarding training intensity, an exercise training session was designated as performed at an adequate intensity when, based on heart rate, at least 3 of the 5 prescribed high-intensity exercise bouts complied with the prescribed intensity, or when the training session intensity reported on the Borg RPE score was equal or higher than prescribed. The number of attended sessions in which the prescribed intensity was accomplished (based on either heart rate or Borg RPE score) was divided by the total number of attended sessions and presented as a percentage. For training time, the observed duration of the sessions was divided by the prescribed duration of the sessions and presented as a percentage. Adherence was deemed adequate if ≥ 80% as assessed individually for training frequency, training intensity, and training time. Participant appreciation of the tele-prehabilitation program, as scored by the patient appreciation questionnaire, and user-friendliness of the mobile phone application of HC@Home, as scored by the systems usability questionnaire, were reported descriptively. A systems usability questionnaire score ≥ 73 was considered good, and a score of ≥ 85 excellent user-friendliness (Bangor et al. 2008). Continuous data representing changes in aerobic fitness during the tele-prehabilitation program were presented as median and interquartile range (IQR). Pre-post analysis was performed using the non-parametric Wilcoxon signed-rank test. A *P*-value < 0.05 was considered statistically significant.

## Results

**Fig. 1:**
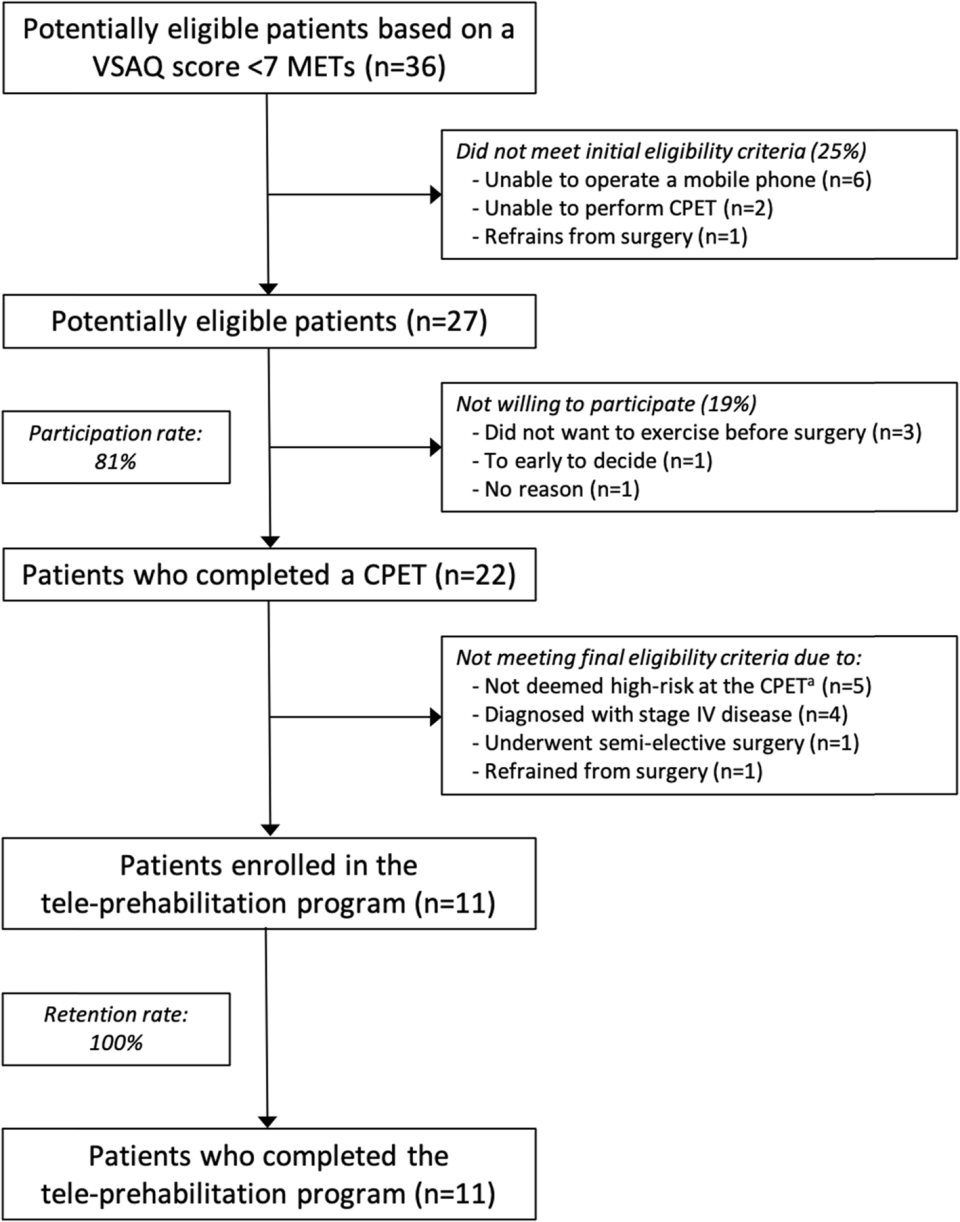
Flowchart of inclusion and participation. ^a^ High-risk is defined as an oxygen uptake (VO_2_) at the ventilatory anaerobic threshold (VAT) ≤ 11 mL/kg/min or at peak exercise (VO_2peak_) ≤ 18 mL/kg/min at the preoperative CPET. Abbreviations: CPET, cardiopulmonary exercise test; METs, metabolic equivalent of task; VSAQ, veterans-specific activity questionnaire

A total of 36 patients were contacted to check for eligibility and willingness to participate. The participation rate was 81%. Eventually, a total of 11 patients were eligible and were enrolled in the tele-prehabilitation program. Reasons for non-willingness or inability to participate and reasons for exclusion are depicted in Fig. 1. Baseline characteristics of the included participants are listed in Table 1. There were no adverse events or dropouts as a result of the tele-prehabilitation program. One participant failed to perform the post-prehabilitation assessment and one patient was unable to perform adequately on the constant work rate test, both due to a feeling of general discomfort and nausea on the day of assessment.

**Table 1:** Participant characteristics (*n* = 11)

Characteristics	Included *n* = 11
Age (years)	74 [68–78]
Sex ratio (male; female)	6;5
Living status	
Living alone	*n* = 5 (45%)
Living with partner	*n* = 6 (55%)
Body mass index (kg/m^2^)	29.1 [24.6–33.1]
Smoking status	
Never	1 (9%)
Former	9 (82%)
Current	1 (9%)
Age-adjusted comorbidity index	
2–3	1 (9%)
4–5	3 (27%)
6+	7 (64%)
ASA-classification	
I	1 (9%)
II	3 (27%)
III	6 (55%)
IV	1 (9%)
VSAQ (METs)	4 [3–5]
VO_2_ at the VAT (mL/kg/min)	9.3 [7.5–10.0]
VO_2peak_ (mL/kg/min)^a^	14.8 [12.7–15.6]
Hemoglobin level (mmol/L)	7.1 [6.7–8.7]
Albumin levels (g/L)	37 [35–40]
PG-SGA-SF score	
0	5 (45%)
2	2 (18%)
5+	4 (36%)
Tumor location	
Colon	8 (73%)
Rectum	3 (27%)
Tumor stage	
I	5 (46%)
II	3 (27%)
III	3 (27%)
Type of surgery	
Hemicolectomy	8 (73%)
Other	3 (27%)
Surgical approach	
Open	1 (9%)
Laparoscopic	8 (73%)
Endoscopic	1 (9%)
Conversion to open	1 (9%)
Received neoadjuvant treatment	1 (9%)

Data are presented as number of patients (%) or median [IQR], unless stated otherwise

^a^ *n* = 9, as a maximal effort was required based on a respiratory exchange ratio at peak exercise ≥1.10 and/or a heart rate at peak exercise > 85% of predicted

Abbreviations: *ASA* American Society of Anesthesiologists, *MET* metabolic equivalent of task, *PG-SGA-SF* patient-generated subjective global assessment short form, *VAT* ventilatory anaerobic threshold, *VO*_*2*_ oxygen uptake, *VO*_*2peak*_ oxygen uptake at peak exercise, *VSAQ* veterans-specific activity questionnaire

### Tele-prehabilitation program

The median time that elapsed between diagnosis (date of endoscopy) and surgery was 34 days (range 20–51) for participants with surgery as their first treatment (*n* = 10; 91%). Median time between start of the physical exercise training program and surgery was 23 days (range 6–30) (Table 2). Adherence with regard to the tele-prehabilitation program’s training frequency, intensity, and time (FIT) is depicted in Table 2. Combined, the participants performed a total of 109 out of 120 prescribed training sessions (91%). In addition, 9 out of 11 participants (81%) managed to adhere to ≥ 80% of the prescribed sessions. Mean ± SD training intensity reached throughout each entire training session was 78 ± 9% of the maximal heart rate during CPET and a score of 14 ± 1 on the 6-20 Borg RPE scale. Although participants were able to adhere to the prescribed exercise intensity in 84% (91 out of 109) of the performed exercise sessions, only 63% of the participants were able to reach the prescribed intensity in ≥80% of their performed sessions. With regard to exercise session time (duration), all 11 participants managed to perform the prescribed exercise duration in ≥ 80% of the sessions. Duration of the performed physical exercise training sessions of all patients combined was 3475 min (100% of prescribed).

**Table 2:** Performed training session frequency, intensity, and time, adherence, and changes in physical fitness of the physical exercise training module of the tele-prehabilitation program

Participant ID	Frequency	Intensity	Time	Treatment initiation intervals		Change in physical fitness between pre- (***T***_**0**_) and post-prehabilitation (***T***_**1**_) assessment								
	Number of sessions (% of prescribed)	Number of sessions with adequate intensity, number (%)	Combined exercise duration of all sessions, minutes (% of prescribed)	Time from endoscopy to start prehabilitation (days)	Time from start prehabilitation to surgery (days)	Time to exhaustion on the constant work rate test (s)			Repetitions on the 30-s chair-stand test (number)			4-m gait speed test (m/s)		
						*T*_0_	*T*_1_	Change (%)	*T*_0_	*T*_1_	Change (%)	*T*_0_	*T*_1_	Change (%)
1	19 (68%)	19 (100%)	640 (85%)	110^a^	58^a^	180	316	+ 136 (+ 76%)	10	14	+ 4 (+ 40%)	0.7	0.9	+ 0.2 (+ 29%)
2	5 (83%)	5 (100%)	159 (88%)	14	12	306	316	+ 10 (+ 3%)	14	18	+ 4 (+ 29%)	1.1	1.3	+ 0.2 (+ 18%)
3	13 (108%)	9 (69%)	254 (115%)	16	26	303	481	+ 178 (+ 59%)	8	10	+ 2 (+ 25%)	1.0	0.9	− 0.1 (− 10%)
4	10 (91%)	9 (90%)	349 (95%)	12	25	581	773	+ 192 (+ 33%)	13	18	+ 5 (+ 38%)	1.3	1.1	− 0.2 (− 15%)
5	13 (100%)	11 (85%)	449 (112%)	21	30	606^b^	304^b^	− 302 (− 50%)^b^	10^b^	16^b^	+ 6 (+ 60%)^b^	1.3^b^	1.4^b^	−
6	12 (100%)	12 (100%)	407 (113%)	11	28	326	364	+ 38 (+ 6%)	16	17	+ 1 (+ 6%)	1.3	1.2	− 0.1 (− 8%)
7	10 (100%)	7 (70%)	312 (104%)	7	21	260	237	− 23 (− 9%)	10	10	0 (0%)	1.0	–	–
8	6 (100%)	4 (67%)	177 (98%)	14	13	783	657	− 126 (− 16%)	13	16	+ 3 (23%)	1.4	1.6	+ 0.2 (+ 14%)
9	6 (100%)	5 (83%)	183 (102%)	15	16	427	459	+ 32 (+ 8%)	12	15	+ 3 (+ 25%)	1.4	1.6	+ 0.2 (+ 14%)
10	2 (67%)	2 (100%)	68 (76%)	14	6	–	–	–	12	–	–	1.0	–	–
11	13 (100%)	8 (62%)	477 (111%)	12	27	307	668	+ 361 (+ 85%)	19	19	0 (0%)	1.4	1.3	− 0.1 (− 7%)^2^
Total	109 (91%)	91 (84%)	3475 (100%)											
Median				14^c^/14^d^	23^c^/25^d^	317	412	95 (+ 30%)	12	16	4 (+ 33%)	1.3	1.3	0.0 (0%)

^a^Participant received neoadjuvant treatment

^b^Participant had a general feeling of discomfort during post-prehabilitation assessment and therefore did not perform adequately

^c^All participants (*n* = 11)

^d^Excluding the participant receiving neoadjuvant treatment (*n* = 10)

*Abbreviations*: *T*_*0*_ pre-prehabilitation, *T*_*1*_ post-prehabilitation (1 or 2 days before surgery)

Participant appreciation of the tele-prehabilitation program is depicted in Table 3. All participants indicated that the tele-prehabilitation program prepared them well for the surgical intervention.

**Table 3:** Patient appreciation of the tele-prehabilitation program

	Strongly disagree				Strongly agree
	1	2	3	4	5
1. The aim of the intervention in preparation of the surgical treatment was clear to me.	–	–	–	–	11 (100%)
2. The perceived exertion during the cardiopulmonary exercise test was high.	1 (9%)	–	4 (36%)	3 (27%)	3 (27%)
3. In my opinion, the cardiopulmonary exercise test was useful.	–	–	1 (9%)	–	10 (91%)
4. The perceived exertion during the home-based exercises was high.	1 (9%)	1 (9%)	2 (18%)	4 (36%)	3 (27%)
5. In my opinion the home-based exercises were useful.	-	-	-	1 (9%)	10 (91%)
6. I was motivated to perform the home-based exercises.	–	–	–	1 (9%)	10 (91%)
7. I experienced the home-based exercises as pleasant.	–	1 (9%)	1 (9%)	3 (27%)	6 (54%)
8. The home-based exercises were time-consuming.	7 (64%)	2 (18%)	1 (9%)	1 (9%)	-
9. The weekly evaluations by telephone were beneficial to me.	–	–	–	1 (9%)	10 (91%)
10. I experienced it be pleasant to be able to perform the exercises independently at home.	–	–	–	1 (9%)	10 (91%)
11. I think the tele-prehabilitation program prepared me well for the surgical treatment.	–	–	–	2 (18%)	9 (82%)

Data are presented as the number of patients (%)

**Fig. 2:**
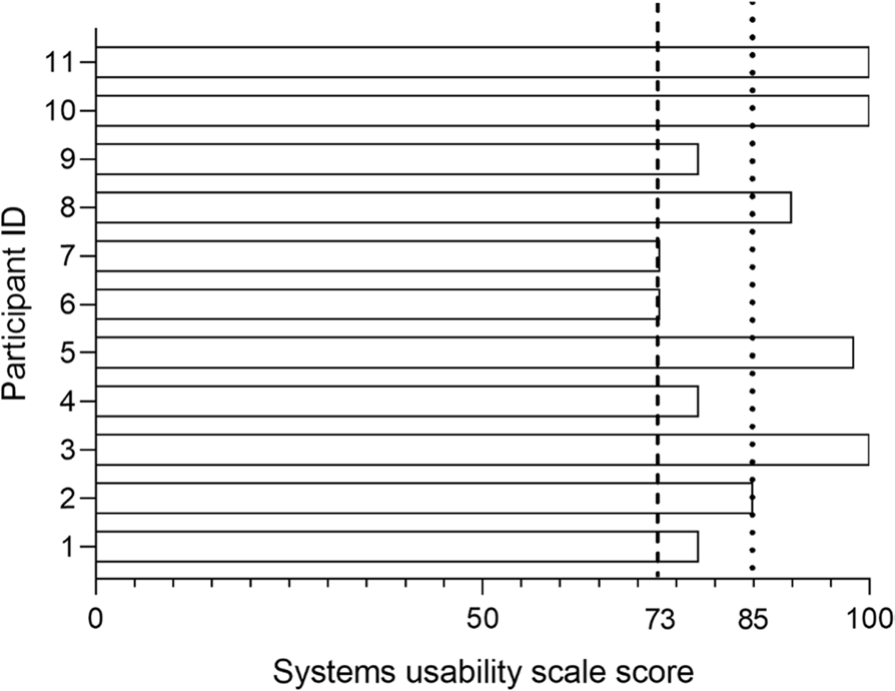
Participant’s individual score on the systems usability scale. Dashed and dotted lines represent thresholds of respectively good (≥ 73) and excellent (≥ 85) usability of the mobile phone application used for tele-prehabilitation

Median systems usability questionnaire score was 85 (IQR 78–100). All 11 patients had a systems usability questionnaire score ≥ 73, indicating that the user-friendliness of the mobile phone application was good. Six patients had a systems usability questionnaire of ≥ 85% indicating excellent user-friendliness (Fig. 2).

**Fig. 3:**
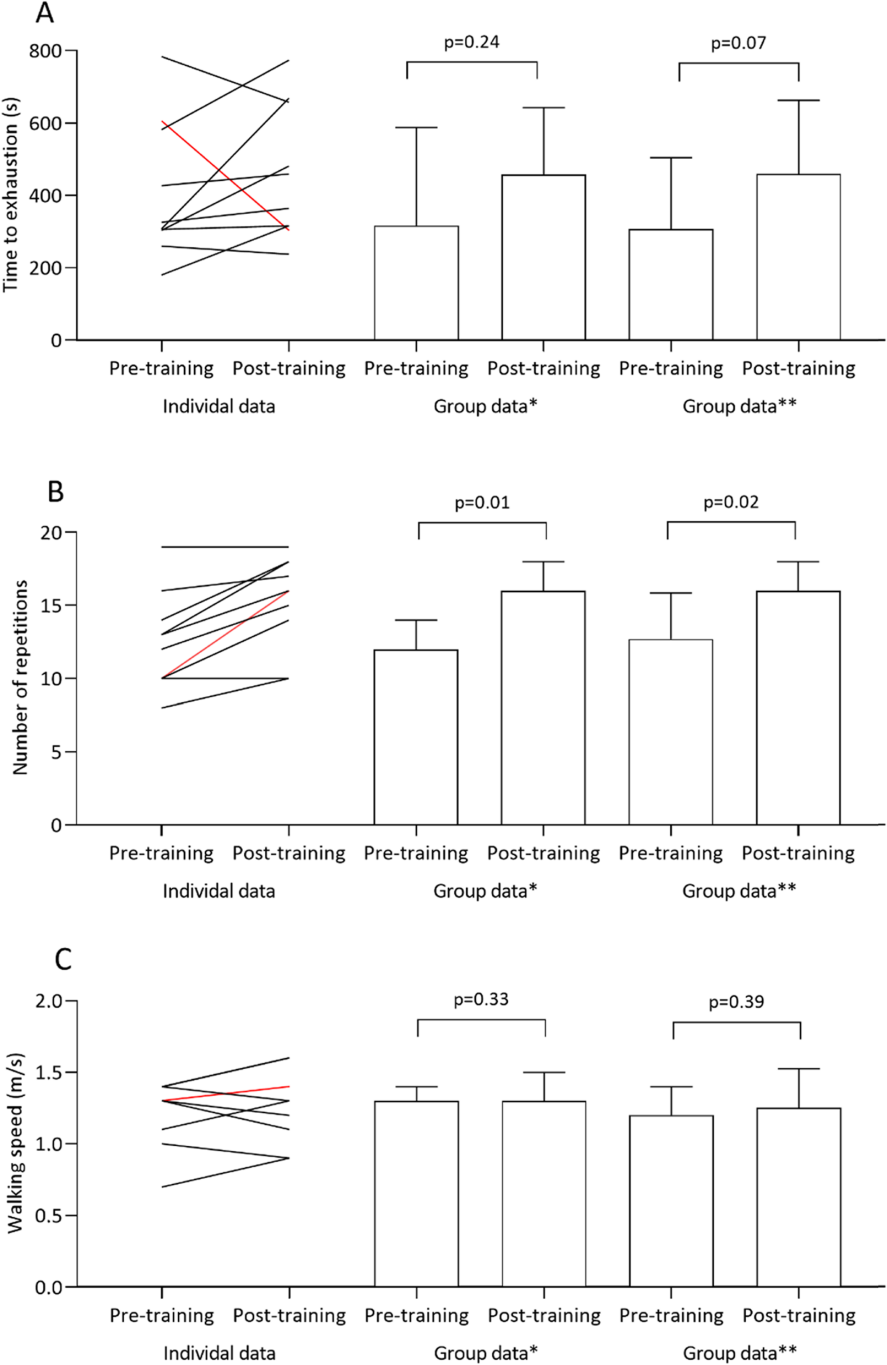
Preliminary changes in aerobic fitness before (pre-prehabilitation) and after (post-prehabilitation) the tele-prehabilitation program. Graphs represent outcomes of the constant work rate test (**A**), the 30-s chair-stand test (**B**) and the 4-m gait speed test (**C**). Both individual data (left) and group data (right) are presented. For the group data, bars indicate median values with error bars representing the interquartile range. *P*-values indicate significance level tested with the non-parametric Wilcoxon signed-rank test. * All patients that completed the post-prehabilitation (*T*_1_) assessment (*n* = 10). ** Excluding the patient that had a general feeling of discomfort during post-prehabilitation (*T*_1_) assessment (*n* = 9), which is highlighted in red (participant ID 5) in the individual data plot

Pre- and post-prehabilitation (preoperative) assessment of physical fitness was performed in 10 participants (91%) as shown in Fig. 3. With regard to time to exhaustion on the constant work rate test, 7 participants (70%) had an equal or longer time to exhaustion at the post-prehabilitation evaluation, whereas 3 patients (30%) had a shorter time to exhaustion. Of these 3 patients, 1 patient was unable to perform adequately on the constant work rate test after the tele-prehabilitation program due to general discomfort and nausea. From pre- to post-prehabilitation, time to exhaustion on the constant work rate test changed from a median of 317 s to a median of 412 s (*p* = 0.24) and from a median of 307 s to a median of 459 s (*p* = 0.07) with and without the participant with a general feeling of discomfort at the post-prehabilitation assessment, respectively. Following the tele-prehabilitation program, the number of repetitions at the 30-s chair-stand test significantly improved from a median of 12 to a median of 16 repetitions (*p* = 0.01). No significant changes were observed in walking speed as measured by means of the 4-min walk test (*p* = 0.33).

## Discussion

The current study aimed to evaluate the feasibility of a home-based tele-prehabilitation program in high-risk patients with colon or rectal cancer scheduled for surgery. Tele-prehabilitation was deemed feasible, as willingness to participate was high (81%) and adherence was good (> 80%). Patients felt the tele-prehabilitation prepared them well for surgery. Changes in physical fitness measured before and after the tele-prehabilitation program showed a trend towards improved physical fitness after tele-prehabilitation. There are no specific recommendations regarding the sample size of feasibility studies. Although only 11 participants were included in the current study, these participants were deemed representative of a larger population of patients with colorectal cancer with a high risk for postoperative complications, because participant characteristics are in line with participant characteristics in a larger randomized controlled trial in the same population (Berkel et al. 2022).

The participation rate of 81% in the current study was comparable to the participation rate in previous tele-prehabilitation programs (Wu et al. 2021; Waller et al. 2021) and a hospital-based (Suen et al. 2021) prehabilitation program (between 68 and 78%), and higher than a community-based (Berkel et al. 2021) prehabilitation program (56%) before major abdominal surgery. A possible explanation for the observed high willingness to participate is that the tele-prehabilitation program was home-based, personalized, and indirectly supervised, thereby maximizing autonomy and lowering the threshold to participate.

One of the main challenges of “classic” (non-tele-monitored) unsupervised home-based prehabilitation is the often observed low exercise session adherence (Thomas et al. 2019). Previous studies have reported lower exercise session adherence in home-based (~ 70%) compared to hospital-based (> 95%) prehabilitation programs (Thomas et al. 2019). Exercise session adherence (exercise frequency) in the current home-based tele-prehabilitation study was high (93% of prescribed) and almost comparable to supervised hospital-based prehabilitation programs (97–99%) (Thomas et al. 2019). Personalization of the tele-prehabilitation program and flexibility concerning planning of training sessions might have contributed to this high exercise session adherence, as autonomy is mentioned as one of the key factors that enable patients to participate in prehabilitation in the stressful and busy period between diagnosis and surgery (Beck et al. 2021). In addition, it has been shown that some kind of supervision is essential for patients in order to stay motivated (Beck et al. 2021). In this regard, home-based tele-prehabilitation might be superior to classic unsupervised home-based prehabilitation as the tele-monitoring in combination with weekly telephone calls might provide sufficient pressure and supervision for patients to keep motivated. In the current study it was noted that participants appreciated the weekly follow-up phone calls and reported them as useful.

Apart from adherence to training frequency alone, full adherence to a physical exercise training program should also be evaluated based on training intensity and training time (Franssen et al. 2022). Although overall adherence to the exercise intensity was > 80%, only 7 participants (63%) managed to adhere to the prescribed intensity in ≥ 80% of the sessions. Exercise intensity is one of the key factors that contribute to the effectiveness to improve aerobic fitness in a short-term physical exercise program (Franssen et al. 2022); therefore, adherence to exercise training intensity needs to be optimized. In the current study, participants performed exercises unsupervised and tele-monitored after an initial home-based introduction session. It was noted that all participants reached the prescribed intensity during the first home-based supervised training session. In the following unsupervised training sessions, adherence to exercise training intensity was less consistent. This could mean that more direct supervision and encouragement are needed to adhere to the exercise intensity. Therefore, adherence concerning exercise intensity might be improved by adding a weekly supervised session (preferably home-based or by using video conferencing) in order to motivate and coach patients to adhere to the exercise program. In addition, direct feedback regarding the physical exercise training session intensity and duration provided by the mobile phone application might be helpful for patients to comply with the prescribed program.

In general, participants appreciated the tele-prehabilitation program. Most participants reported they experienced it pleasurable to perform exercises independently at home, they reported that the weekly telephone calls were helpful and that the tele-prehabilitation program prepared them well for the surgical procedure. In addition, the usefulness of the smart phone application that was used for the tele-prehabilitation program, as rated by the systems usability questionnaire ranged from good to excellent. All participants managed to use the smart phone application independently (or with help of their buddy) after a short introduction session. These results are in accordance with a multimodal tele-prehabilitation study in patients with abdominal cancer that used commercially available wearables to improve physical fitness prior to surgery (Waller et al. 2022). Patients in the latter study (Waller et al. 2022) reported that the wearables were easy to use and motivational to improve physical activity.

The current study has several limitations. Some patients were excluded for reasons that have to do with feasibility of tele-prehabilitation, such as being unable to operate a mobile phone (*n* = 6, 17%) or being unable to perform a CPET (*n* = 2, 6%). Although these excluded patients did not undergo CPET and therefore it is uncertain whether they would have been classified as high risk, the reasons for exclusion are specific for a tele-prehabilitation program. In addition, previous research has shown that patients that are unable to perform CPET should be treated as high-risk (Lai et al. 2013).

A major obstacle for the implementation of prehabilitation is the short diagnosis-to-surgery interval (median of 34 days, range 20–51 days). Combined with a relatively long interval between diagnosis and start of the prehabilitation (median of 14 days, range 7–21 days), this leaves limited time for a comprehensive prehabilitation program in patients who receive surgery as their first treatment. These time limitations are often caused by logistics (e.g., delayed final diagnosis and/or surgical planning) and time constraints. Efforts should be made to use the available time as efficiently as possible, for example by starting screening, assessment, and prehabilitation directly after colorectal cancer diagnosis by endoscopy. Nevertheless, although essential for effective prehabilitation, these strict time constraints are not strongly supported by evidence (Franssen et al. 2021a; Strous et al. 2019; Molenaar et al. 2021) and not specific towards tele-prehabilitation, but involve a broader problem that is generally seen in prehabilitation studies (Boereboom et al. 2019). Another limitation that was observed in the current study was that in 3 participants (27%) heart rate could not be used as an indicator of exercise intensity due to chronotropic incompetence. Although this was partly covered by the use of the Borg RPE score, especially in non-real-time monitored interventions such as tele-prehabilitation, the combination of perceived effort (i.e., Borg RPE) with a form of objective monitoring is of major importance due to the lack of direct supervision. Accelerometer-based (Arvidsson et al. 2019; Jung et al. 2015) or respiratory rate monitoring (Nicolo et al. 2017) might be alternative measures that can be used in addition to heart rate monitoring to provide an objective estimate of exercise intensity when heart rate monitoring is not feasible (e.g., participants with severe cardiac arrhythmia or chronotropic incompetence).

Strengths of the current tele-prehabilitation program are that the intervention was personalized and focused on high-risk patients, based on the CPET, and the use of an exercise intervention blueprint meaning the exercise intervention was adjusted based on the participant’s preferences and characteristics. In addition, instead of attendance rates only, the current study reported full adherence (frequency, intensity, and time) to the physical exercise intervention. Another strength of the current study is that tele-prehabilitation was implemented within the current colorectal cancer treatment pathway and no additional study visits were required for participants.

In future research, attempts should be made to optimize adherence to the exercise training intensity of the tele-monitored physical exercise program, for example by using a combination of physically supervised and tele-monitored supervision. Furthermore, a larger prospective observational study could be designed to evaluate willingness to participate, adherence, and (cost-)effectiveness of prehabilitation when different forms of multimodal prehabilitation (e.g., tele-prehabilitation, community-based prehabilitation, and hospital-based prehabilitation) are presented to patients.

## Conclusion

Results of this feasibility study have shown that a home-based tele-prehabilitation program is feasible and appreciated in high-risk patients undergoing surgery for colorectal cancer. However, efforts should be made to further improve adherence towards exercise intensity. More research is needed to establish the (cost-)effectiveness of tele-prehabilitation with regard to improvements in preoperative aerobic fitness and reduction of postoperative complications before definitive conclusions can be drawn.

## Data Availability

The datasets used and/or analyzed during the current study are available from the corresponding author on reasonable request.

## Abbreviations

CPET: Cardiopulmonary exercise test
IQR: Interquartile range
RPE: Rating of perceived exertion
SD: Standard deviation of the mean
*T*_0_: Pre-prehabilitation
*T*_1_: Post-prehabilitation (1 or 2 days before surgery)
VAT: Ventilatory anaerobic threshold
VO_2peak_: Oxygen uptake at peak exercise
